# Tailored Prompting to Improve Adherence to Image-Based Dietary Assessment: Mixed Methods Study

**DOI:** 10.2196/52074

**Published:** 2024-04-15

**Authors:** Lachlan Lee, Rosemary Hall, James Stanley, Jeremy Krebs

**Affiliations:** 1 Department of Medicine, University of Otago, Wellington, New Zealand; 2 Biostatistics Group, University of Otago, Wellington, New Zealand

**Keywords:** dietary assessment, diet, dietary, nutrition, mobile phone apps, image-based dietary assessment, nutritional epidemiology, mHealth, mobile health, app, apps, applications, applications, image, RCT, randomized, controlled trial, controlled trials, cross-over, images, photo, photographs, photos, photograph, assessment, prompt, prompts, nudge, nudges, food, meal, meals, consumption, behaviour change, behavior change

## Abstract

**Background:**

Accurately assessing an individual’s diet is vital in the management of personal nutrition and in the study of the effect of diet on health. Despite its importance, the tools available for dietary assessment remain either too imprecise, expensive, or burdensome for clinical or research use. Image-based methods offer a potential new tool to improve the reliability and accessibility of dietary assessment. Though promising, image-based methods are sensitive to adherence, as images cannot be captured from meals that have already been consumed. Adherence to image-based methods may be improved with appropriately timed prompting via text message.

**Objective:**

This study aimed to quantitatively examine the effect of prompt timing on adherence to an image-based dietary record and qualitatively explore the participant experience of dietary assessment in order to inform the design of a novel image-based dietary assessment tool.

**Methods:**

This study used a randomized crossover design to examine the intraindividual effect of 3 prompt settings on the number of images captured in an image-based dietary record. The prompt settings were control, where no prompts were sent; standard, where prompts were sent at 7:15 AM, 11:15 AM, and 5:15 PM for every participant; and tailored, where prompt timing was tailored to habitual meal times for each participant. Participants completed a text-based dietary record at baseline to determine the timing of tailored prompts. Participants were randomized to 1 of 6 study sequences, each with a unique order of the 3 prompt settings, with each 3-day image-based dietary record separated by a washout period of at least 7 days. The qualitative component comprised semistructured interviews and questionnaires exploring the experience of dietary assessment.

**Results:**

A total of 37 people were recruited, and 30 participants (11 male, 19 female; mean age 30, SD 10.8 years), completed all image-based dietary records. The image rate increased by 0.83 images per day in the standard setting compared to control (*P=.*23) and increased by 1.78 images per day in the tailored setting compared to control *(P*≤.001). We found that 13/21 (62%) of participants preferred to use the image-based dietary record versus the text-based dietary record but reported method-specific challenges with each method, particularly the inability to record via an image after a meal had been consumed.

**Conclusions:**

Tailored prompting improves adherence to image-based dietary assessment. Future image-based dietary assessment tools should use tailored prompting and offer both image-based and written input options to improve record completeness.

## Introduction

Accurate assessment of dietary intake is an essential component in the study of nutrition, energy balance, and interventions for obesity and diabetes. Current tools to measure dietary intake are either too imprecise, or they are precise but too expensive, to provide an accurate assessment for clinical use or large population studies [1].

An increasingly popular tool for measuring dietary intake is image-based dietary assessment. Image-based methods are characterized by images rather than text as the main form of data input and have been reported as a preferred method, particularly among children and adolescents [2]. Image-based dietary assessment tools, such as Easy Diet Diary, MealLogger, and MyFitnessPal, are increasingly being adopted for clinical, research, and personal use [3-5]. The accuracy of image-based methods is comparable to traditional text-based methods; in a meta-analysis by Ho et al [6], image-based methods underreported energy intake by 20% compared to doubly labeled water, the gold standard for measuring energy intake. For comparison, Burrows et al [7] showed that text-based food records underreported energy intake by 11% to 41% compared to doubly labeled water.

Innovations in phone cameras and computer vision, alongside widespread smartphone ownership, provide an opportunity to advance image-based dietary assessment [8]. For image-based methods to achieve high accuracy, they must improve how users capture the content and portion sizes of the foods they eat. The most basic challenge is that users must remember to record in real time, for the simple reason that they cannot photograph food that has already been eaten. Reminding users to capture images using customized text prompts immediately before a meal has previously been shown to improve adherence to an image-based method [9].

This mixed methods study used a randomized crossover trial to examine if the timing of customized text prompts affected the completeness of dietary recording using an image-based method; we also performed a qualitative study of participant attitudes toward image- and text-based dietary assessment methods and text prompting. This study and its findings will inform the design of a novel dietary assessment application.

## Methods

### Recruitment

Participants were recruited through a circulating poster on university staff and student email lists, social media sites, and word of mouth. Potential participants contacted the researchers, who then provided information about the study. Individuals were eligible if they met the following inclusion criteria: they (1) were aged ≥18 years, (2) had access to a smartphone with a camera, (3) had internet access on their mobile device, and (4) could speak and read English. There were no additional exclusion criteria.

### Ethical Considerations

This study was approved by the University of Otago Human Ethics Committee (H21/049). Informed consent was required to participate in the study and was gained at the screening visit. Participants could opt out at any time during the study and were not required to provide a rationale. All participant data were deidentified. Each participant was compensated with a NZ $50 (US $30) supermarket voucher.

### Randomized Crossover Trial

**Figure 1.**
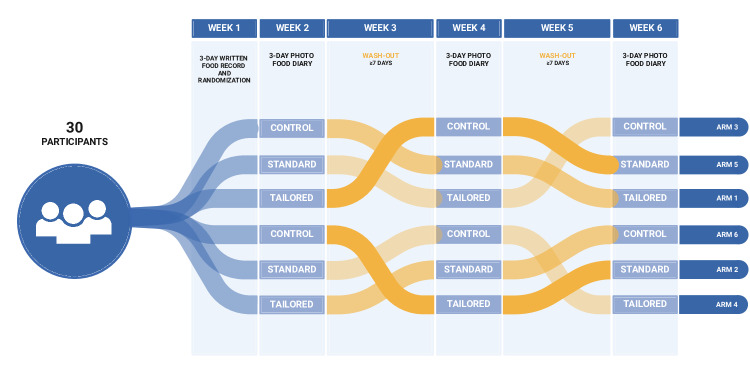
Overview of randomized crossover trial design of text prompt settings. Control setting: no prompts received; standard setting: prompts received at 7:15 AM, 11:15 AM, and 5:15 PM; tailored setting: prompts received at times specific to participants’ typical meal times as recorded with a text-based dietary record.

The randomized crossover trial compared standard and tailored text prompts to a control period without prompts while completing an image-based dietary record. The study design is shown in Figure 1. Participants attended a screening visit at the research center at the University of Otago, Wellington, New Zealand. After providing written informed consent, eligible participants completed baseline measurements: (1) height in meters using a wall-mounted stadiometer to the nearest 0.5 cm, (2) weight in kilograms to the nearest 0.1 kg using an electronic scale (TBF-300; Tanita Corporation), and (3) resting metabolic rate in kilojoules per day using a ventilated hood (PromethION High-Definition Room Calorimetry System; Sable Systems International). Participants were shown how to use the Easy Diet Diary app (Xyris Software) for the text-based dietary record and image-based dietary record and given the opportunity to ask questions. Easy Diet Diary is free to download from the iOS and Android app stores. Participants then recorded all dietary intake and meal times for 3 days in a text-based dietary record using the text-based features of Easy Diet Diary. Participants were contacted by a researcher if no food was recorded that day to confirm the accuracy of the text-based dietary record. The meal times from this first text-based dietary record were used to calibrate the timing of tailored text prompts for use in the subsequent randomized part of the trial.

After recording the text-based dietary record, participants were randomized into 1 of 6 arms, each arm having a unique order of the 3 text prompt conditions, as described in Figure 1. Participants were reminded to begin their 3-day image-based dietary records using a text message that required confirmation. Each of the image-based dietary records was recorded over 2 weekdays and 1 weekend day. After each 3-day image-based dietary record, participants underwent a washout period of at least 7 days of no recording to mitigate fatigue or training effects. This process was repeated until each participant completed recording under all 3 conditions.

Under the control setting, participants received no prompts. For the standard condition, text prompts were sent at 7:15 AM, 11:15 AM, and 5:15 PM. Data on the typical meal times in New Zealand were unavailable, so these times were chosen to capture participants who might have early meal times. Individually tailored text prompts were determined based on the meal timing provided during the initial text-based dietary record. Each meal time in the text-based dietary record was recorded via the Notes feature of Easy Diet Diary using the following format: “[Time of eating] – [Description of food item consumed],” for example, “1.50 PM – Salmon bagels.” Tailored text prompts were set according to the following guidelines: (1) prompts were sent 15 minutes before the earliest recorded eating episode and (2) a prompt for snacks was sent if snack intakes occurred within 1 hour of each other on 2 of 3 days during the text-based dietary record. Both standard and tailored text prompts read, “This is a reminder to record your photo food diary using the Easy Diet Diary app.”

### Image Rate

The number of images per participant per day was counted across the predefined recording dates. Images generated by the user were automatically uploaded to Easy Diet Diary Connect (Xyris Software) for image rate analysis at the end of each image-based dietary recording period. No analysis or interpretation of nutritional content in the image was performed.

### Qualitative Methods

The objective of the qualitative component of the study was to explore the participant experience with each text prompt setting, with the purpose of informing the design of future image-based dietary assessment tools. The qualitative component was composed of interviews and questionnaires. All participants were invited to semistructured open-ended interviews that were conducted one-on-one by a single researcher face-to-face or via video conference (Zoom Video Communications, Inc) after the completion of the image-based dietary records. Interviews lasted for 30 to 60 minutes and focused on (1) the experience of diet monitoring, (2) attitudes toward the Easy Diet Diary app, (3) attitudes toward text prompting, and (4) discussing the participant’s “ideal diet monitoring tool.” Transcripts of the interviews were coded and thematically analyzed using NVivo Qualitative Data Analysis (QSR International). Additional online questionnaires hosted on Survey Monkey (Momentive Inc) explored the same topics using Likert scales and rankings to identify participant preferences for dietary assessment methods and text prompt settings.

### Statistical Analysis

#### Overview

Statistical analyses were conducted using R (version 4.2; R Foundation for Statistical Computing). Linear mixed effects analysis of the impact of prompt setting on image rate was conducted using the *lme4* package (Bates, Mächler, Bolker, and Walker). Prompt setting and order were fixed effects within the model—order was included to account for uneven sizes in the 6 different orders. Participants were accounted for as random effects (random intercept model), accounting for repeat observations under each prompt setting and correlation of participant responses across conditions. *P* values for the fixed effect of prompt setting order were obtained using the Wald type 3 test. Q-Q plots were visually inspected and a histogram of the model residuals was checked for homogeneity of variance or any obvious deviations from normality.

#### Power

Previous use of image-based dietary records has seen an image rate of 1.4 images per 2.6 meals: a rate of 54% [10]. From this, we estimated under the control setting a mean of 1.6 images per day (54% capture rate applied across 3 meals per day, excluding snacks). With our intervention, we aimed to be able to detect 2.4 images per day (representing 80% coverage) of meals. A sample size of 20 in each of the 3 measurement conditions had over 80% power to detect this difference in rates with a 2-sided α of 5%. The crossover design improved power to detect differences in adherence rates compared to a between-subjects design.

## Results

### Participants

**Figure 2.**
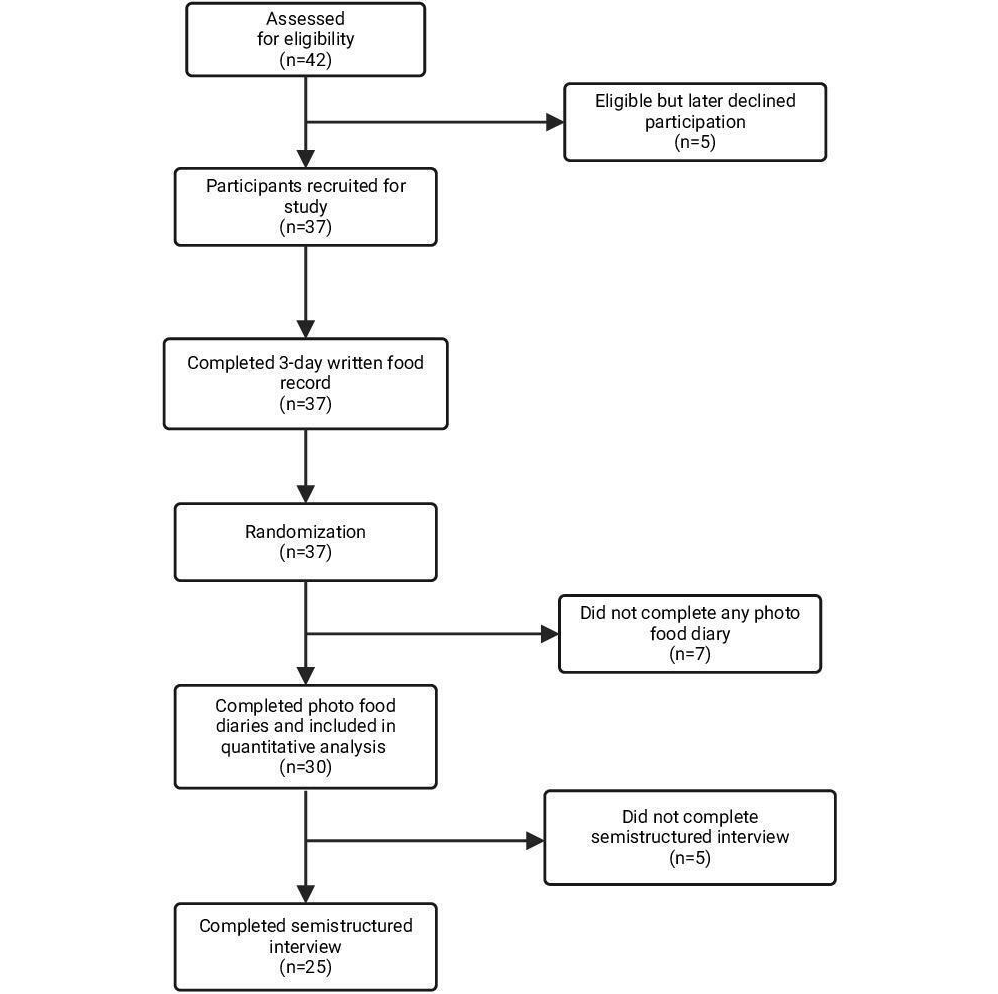
Flow diagram of participants throughout the study.

A total of 42 people were screened for eligibility: 37 met the inclusion criteria and were enrolled, 30 completed the food records, and 25 participated in the qualitative interviews. Seven participants were lost to follow-up and could not be contacted. Five participants did not wish to participate in interviews because of time. A flow diagram of participation is shown in Figure 2.

The number of participants in each arm and their baseline characteristics are shown in Table 1. The prompt setting order can be seen in Figure 1. Arm population sizes were uneven due to asymmetrical loss to follow-up after randomization; each of these participants were categorized as lost to follow-up after failing to respond to 3 emails sent to confirm their ongoing participation in the study prior to starting the active part of the intervention. Only those participants completing all 3 image-based dietary records were included in the analysis.

**Table 1.** Characteristics of participants who completed the image-based dietary records (n=30).

Characteristics		Values
Age (years), mean (SD)		30 (10.8)
BMI (kg/m^2^), mean (SD)		24.6 (5.6)
**Sex, n (%)**		
	Male	11 (37)
	Female	19 (63)
**Ethnicity (self-reported), n (%)**		
	New Zealand European	15 (50)
	Māori	9 (30)
	Pacific	4 (13)
	Asian	1 (3)
	Other European	1 (3)
**Level of education, n (%)**		
	Prefer not to say	1 (3)
	No formal education	1 (3)
	High school graduate	3 (10)
	Current tertiary student	17 (57)
	Tertiary graduate	8 (27)
**Study arm allocation, n (%)** ^**a**^		
	Arm 1	7 (23)
	Arm 2	8 (27)
	Arm 3	4 (13)
	Arm 4	4 (13)
	Arm 5	4 (13)
	Arm 6	3 (10)

a Refer to Figure 1 for text prompt setting order by arm.

### Image Rate

**Figure 3.**
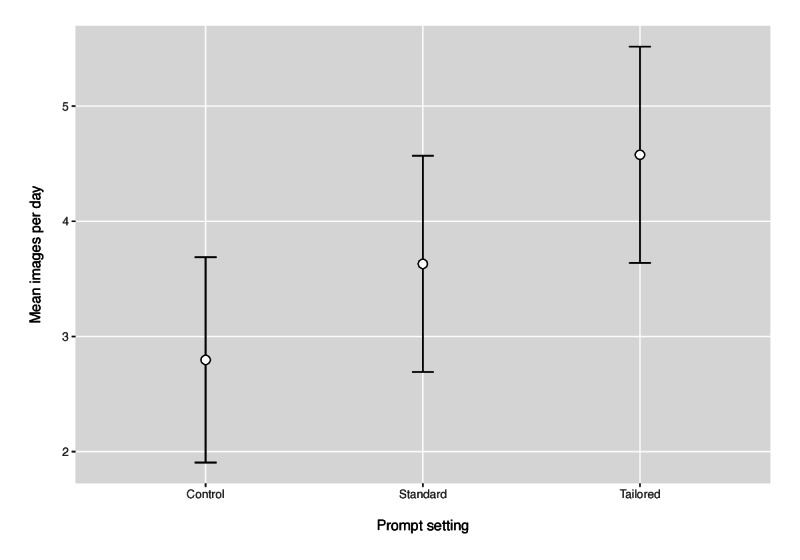
Interval plot of mean image rate (whiskers represent the 95% CI) under each prompt setting.

The image rate, that is, the mean number of images per day recorded on the image-based dietary record, under each prompt setting is shown in Figure 3 with 95% CIs. In the control setting, participants took a mean of 2.8 (95% CI 1.91-3.69) images per day, 3.6 (95% CI 2.69-4.56) images in the standard prompt setting, and 4.6 (95% CI 3.64-5.52) images in the tailored prompt setting. This represents an increase in image rate of 0.83 (95% CI 0.2-2.58) in the standard setting compared to control and an increase of 1.78 (95% CI 1.15-5.51) in the tailored setting compared to control. The order within the sequence of prompt settings had a significant effect *(P*<.001), where the second recording period had 0.82 fewer images per day and the third recording period had 1.19 fewer images per day compared to the first recording.

### Interviews and Questionnaires

Twenty-five participants took part in the interviews (characteristics in Multimedia Appendix 1). Themes that emerged from the interviews were attitudes toward (1) the image-based dietary record, (2) the text-based dietary record, and (3) text prompts. Illustrative quotes relating to these themes can be found in Table 2.

**Table 2.** Themes, subthemes, and illustrative quotes from interviews.

Theme and subtheme		Illustrative quote
**Attitudes toward text-based dietary records**		
	High level of input required	“… the extra time involved, and finding all the random little bits that you’ve put in to make a meal, and then trying to put the quantities in that you’ve eaten… it’s just the time, even though it’s only a minute or two… it’s enough to be a barrier”
	Convenience of retrospective recording	“Because often it would be like you’d get a text to remind you to take a photo, and then you’d forget straight away and with a photo, you can’t eat your food… whereas like, with a written record, that you still know what you’ve eaten. It could just be like, you can write it the end of the day, or like, whenever you remember” “As annoying as it was to kind of write everything out, it meant that I could do it at the end of the day and didn’t have to remember at the time I was eating. For the most part would write it as I was eating to make sure I was… capturing everything that I was eating, but often actually, by the end of the day, I’d be like, I actually had this snack at this time. So I’ll check that in now… it gave me a bit more freedom. But then the picture one was really easy, because you just took a picture… but relied on you to remember at the time you’re eating”
	Challenges with the available database and portion input options	“That’s my main complaint… [not having New Zealand brands]… because then you have to manually put it in” “It would be like in grams or mLs or whatever. And I just kind of put like a handful of something or a scoop of something… kind of tedious trying to be like how many grams would that have been? Or what was it, half a cup or a cup? I guess it’s just because I’m not used to it” “… but in something like Weetbix, it’s fine… to quantify how much how much milk I was using in terms of mLs, which is something that I’m not used to measuring… and even Weetbix, because if I had an odd, an odd number of WeetBix, you can only put everything into two sets of Weetbix. So if you had five, you’re going to put six or four”
**Attitudes toward image-based dietary records**		
	Convenience	“I found photos quite easy, because you just… take a photo and it’s done”
	Less useful data output	“… the fact that you just take a photo of it, but it doesn’t come up with any information… would it be nice if like, you take a photo and all the information comes out and you can kind of track it” “… but I don’t feel like it did anything really? For like purpose of losing weight. … because I took the picture and didn’t tell me like how many calories I was eating… like that cheese and garlic twist, wagyu patty thing… I take picture and think it’s not too bad but not realizing it was 1700 calories”
	Difficulty with habit formation	“Because I wasn’t doing it consistently throughout the whole period, it was a lot harder to do than I think would have been”
	Perceived as more accurate than the text-based dietary record	“[On photo food diaries] … more accurate. You know, this is literally what I’m eating. Whereas doing [the written food record] is sort of like… ‘yeah about a cup of raspberries and I don’t know what the bun was…’ but was that the right one? Or was it like twice as much was this”
	Perceived as less accurate than the text-based dietary record	“I might’ve had 12 eggs. You can’t really tell in my pictures” “There was sometimes where that I’d only had about half my muesli in the morning… I was in a rush, but I would have taken a photo of the whole thing. That would have been half the amount of calories”
**Attitudes toward text prompts**		
	Helpful	“I think that the reminders definitely helped me to remember to take photos. But I think if it was coming from the app, it would have been less annoying”
	Annoying	“They were annoying. The first couple were fine. But towards the end, I literally just ignored all of them” “The days where they were just not at all related to when I usually ate they weren’t really helpful at all, because I would just forget again by the time it came to actually take a picture of my food”

#### Theme 1: Text-Based Dietary Record

Participants reported that the text-based dietary record required a high level of input; this produced detailed food logs with per-item and daily calorie counts, which participants viewed as more useful than the simple image output of the image-based dietary record. Another benefit was the ability to retrospectively record food items; participants often filled in the text-based dietary record at the end of the day—an impossibility with the image-based dietary record. However, participants found completing the text-based dietary record burdensome due to the level of input required: searching and selecting food items, estimating quantities, and reconciling differences between the food item and the database available. The database was often missing food items that were consumed, forcing the participants to input similar but not identical food items. The options available to input portion sizes were also a challenge—participants not knowing the weight of their food in grams, for instance.

#### Theme 2: Image-Based Dietary Record

Participants preferred to use the image-based dietary record; on a Likert scale with 0 indicating preference for text-based dietary records and 100 indicating preference for image-based dietary records, the average response was 60 (SD 32). Most participants found adhering to the image-based dietary record challenging; 83% of interviewed participants reported difficulty in remembering to capture an image of the entire meal. Most cited simple forgetfulness, though some noted difficulty forming the habit of recording with only 3 days of recording interrupted by washout periods. Participants reported difficulty with the image-based dietary record if they had partially or entirely consumed the meal before recording, as they could not capture the consumed meal. Participants reported that the images were simple and convenient to capture but provided no calorie counts or dietary information.

#### Theme 3: Prompts

Attitudes toward the text prompts covered the spectrum from “helpful” to “annoying.” Sending prompts via text messaging was reported as an annoyance by all interviewed participants. These notifications, although welcome in the context of text communication, were intrusive as image-based dietary record reminders; “in-app” reminders were preferred. Despite these annoyances, participants generally found the prompts helpful, with tailored prompts viewed as more helpful.

## Discussion

### Principal Results

This randomized controlled crossover trial demonstrated that text prompts tailored to an individual’s typical meal times improved the completeness of image-based dietary records. This suggests that individual tailoring of prompts will improve accuracy of energy intake assessments. Any prompting was better than no prompts, and tailored prompts were the most effective.

These findings are consistent with work shown in Martin et al [9], where standard and customized prompts were compared in a 2-arm study without crossover or a no-prompt arm. Prompt timing could be improved further by longer calibration periods to determine the individual’s usual meal time and by recording the time of imaging, which was unavailable using Easy Diet Diary. Automated methods of determining prompt timing may provide pragmatic and scalable methods for tailored prompting [11]. Prompting is not unique to dietary assessment, however, and a diverse range of fields use prompts and study their effectiveness [12]. Previous work has identified that an individual’s responsiveness—that is, the likelihood an individual will interact with a prompt or notification—varies based on the characteristics of the person and the prompt [13,14]. Specifically, varying responsiveness to prompts over the course of the day reflects our finding that the timing of a prompt affects the likelihood of interaction. Variable responsiveness poses a particular problem for dietary assessment, where the objective is not only response with a dietary record, but for that response to be accurate. Accuracy of recall has been shown to improve when the recall event is closer to the eating event; that is, recall accuracy decreases with time [15,16]. A prompt must therefore balance the chance of an individual responding to the prompt and the decaying accuracy of the prompted response. Our findings suggest that most individuals in this study were more responsive to dietary assessment prompts closer to meal times, although previous work on prompt responsiveness suggests that this varies from person to person [17]. For some individuals, tailored prompting may reduce the responsiveness of a dietary record. Regular meal times are not the only method to tailor prompts. The use of geolocation to detect likely eating locations, such as restaurants, accelerometers to detect eating or cooking motions, or acoustic sensors to detect chewing noises, among other “context-aware” methods, could also improve prompt timing and appear to be socially acceptable [18-20]. These methods should be selective in their implementation to avoid an excessive number of prompts to participants.

Our findings indicate that prompting tailored to an individual’s food intake pattern improves image-based dietary record completeness. An alternate explanation to consider is that the number of prompts, rather than the timing, improves the image rate. This was not supported by these data. Participants in the tailored setting received a mean of 3.1 prompts per day, while all those in the standard setting received 3 prompts. The effect of tailored prompts on image rate therefore should be attributable to the timing, rather than the number, of prompts. The observed increase of 1.78 images per day with tailored prompts (vs control) is important because this represents mean capture of almost 2 meals across a day that would otherwise have been missed and therefore increases accuracy of dietary intake assessment.

Image recording during the second and third instance of the 3-day image-based dietary record decreased by 0.82 and 1.19 images per day, respectively, compared to the first diary in the sequence. This suggests a fatigue effect that was not mitigated by the 7-day washout period and is of concern where ongoing use of a prompted image-based dietary record is desired in a clinical or research setting. Decreasing energy intake over a recording period has previously been identified in national nutrition surveys such as the United Kingdom’s National Diet and Nutrition Survey. The average decrease from day 1 to 4 was small at 164 kJ, though this may reflect a true decrease in energy intake under observation [21,22]. This fatigue effect has also been found to apply to prompts themselves, with one study of a diet app finding decreasing responsiveness to push notifications over time [17].

### Participant Experience

Participant attitudes toward the image-based dietary record and text-based dietary record contrasted each other. Recording with the image-based dietary record was more convenient at the cost of less useful output in the absence of caloric or macronutrient data. Useful nutritional data can potentially be generated from image-based methods using computer vision–machine learning software, a rapidly advancing field [23]. Text-based dietary records and automated recognition both rely on a representative nutritional database. An incomplete database was highlighted as a barrier to participants providing complete and accurate information. Missing food items or specific brand-name items and inflexible or unfamiliar portion options were challenging. Ensuring that nutritional databases reflect modern and traditional diets, as well as local and culturally relevant foods, should be a priority for current and future dietary assessment tools.

Participants highlighted the convenience of retrospective recording with text-based dietary records, which is not possible with image-based dietary records. The higher number of days with at least 1 entry in the text-based dietary record is suggestive of this retrospective recording, noted in participant interviews and illustrated in Table 2.

Overall, participants reported preference for the image-based dietary record compared to the text-based dietary record, a finding consistent with prior work [24-26]. This preference is notable, as the image-based dietary record did not provide nutritional feedback, and the app was not designed to use images as the primary input method. Participants highlighted that even simple image-based recordings that lack energy or macronutrient intake data provide benefit in the self-assessment of dietary intake, a benefit unique to image-based input that has been identified previously [27,28]. These findings suggest that image-based dietary assessment apps should feature both text and image inputs, providing users the convenience of both simple image-based recording and retrospective text-based recording. Finally, participants preferred tailored prompts and indicated a preference for in-app prompting versus text message prompts.

### Strengths

This study had several strengths. The crossover design allowed for intraindividual assessment. Instructing participants to only record images during the image-based dietary records in the absence of automation to provide numeric caloric or macronutrient data means that this information was not available to participants, but this does more closely reflect participant behavior using an automated image-based dietary assessment tool. The diversity of participants included in this study is a strength, with representation of Māori and Pacific views on dietary assessment, which is particularly important in New Zealand.

### Limitations

This study had a number of limitations. A 3-day text-based dietary record and rudimentary guidelines were used to generate tailored prompt timing; future studies should use more sophisticated methods—such as machine learning techniques—and expanded timeframes to generate tailored prompt timing. The 3-day time frame for the text-based dietary record and each of the image-based dietary records is brief, and future studies should ideally extend to longer time frames; however, extending these time frames must be balanced against the decreasing accuracy over time. This study placed an artificial dichotomy on the method of dietary assessment. In reality, individuals are free to alternate between image-based and text-based input; a key finding of this study is the importance of offering both, and there are many platforms that provide both methods. We did not directly compare the accuracy of image-based versus text-based input using manual or automated analysis of the images to determine if one method produces more reliable energy intake, macronutrient, or micronutrient estimates, which would affect the interpretation of dietary assessment data from platforms that use both methods.

### Conclusions

Individualized tailoring of prompts to match likely meal times improved the completeness of image-based dietary record entries. This is important to improve the accuracy of dietary intake assessments in both clinical and research settings. Although participants preferred the image-based diary, they also identified the need to be able to retrospectively enter data for meals that they had missed recording with an image. The accurate analysis of nutritional composition of a meal from an image requires interpretation of the volume and composition of a meal and integration with nutrient data. Currently available nutritional databases have limitations in the items included, particularly in certain population groups and for culturally specific foods. Therefore, we propose 3 recommendations for future iterations of image-based dietary assessment apps: (1) allowing options for both image-based and written input, (2) publishing and continually updating a nutritional database relevant to the population under study, and (3) the use of tailored in-app prompting for both prospective recording and retrospective recording of missed food items.

## Supplementary material

10.2196/52074 Multimedia Appendix 1 Demographics of interviewed participants.
