# Supplementary material for: Tailored Prompting to Improve Adherence to Image-Based Dietary Assessment: Mixed Methods Study
Source: JMIR Mhealth Uhealth. 2024 Apr 15;12:e52074. doi: 10.2196/52074 (PMC11034420; doi:10.2196/52074)
Supplement: Multimedia Appendix 1 [file mhealth-v12-e52074-s001.pdf]

Multi-media Appendix (1): demographics of interviewed participants.

Table 1. Demographics of interviewed participants (n=25)

	Characteristics	n (%)
**Age**		
	18-25	17 (68%)
	26-30	4 (16%)
	30+	4 (16%)
**Sex**		
	Male	11 (44%)
	Female	14 (56%)
	Intersex	0 (0%)
	Unknown	0 (0%)
**Ethnicity**		
	New Zealand European	14 (56%)
	Māori	5 (20%)
	Pacific	4 (16%)
	Asian	1 (4%)
	Other	1 (4%)
**BMI**		
	Underweight (<20)	1 (4%)
	Healthy (20–24.9)	17 (68%)
	Overweight (25–29.9)	5 (20%)
	Obese (≥ 30)	2 (8%)
**Level of Education**		
	No formal qualification	0 (0%)
	High school graduate	3 (12%)
	Current tertiary student	16 (64%)
	Tertiary graduate	5 (20%)
	Prefer not to say	1 (4%)
